# Neural substrates and behavioral profiles of romantic jealousy and its temporal dynamics

**DOI:** 10.1038/srep27469

**Published:** 2016-06-07

**Authors:** Yan Sun, Hongbo Yu, Jie Chen, Jie Liang, Lin Lu, Xiaolin Zhou, Jie Shi

**Affiliations:** 1 National Institute on Drug Dependence, Peking University, Beijing 100191, China; 2 Center for Brain and Cognitive Sciences and Department of Psychology, Peking University, Beijing 100871, China; 3 Department of Pharmacology, School of Basic Medical Sciences, Peking University Health Science Center, Beijing 100191, China; 4 Institute of Mental Health/Peking University Sixth Hospital and National Clinical Research Center for Mental Disorders & Key Laboratory of Mental Health, Peking University, Beijing 100191, China; 5 Peking-Tsinghua Center for Life Sciences and PKU-IDG/McGovern Institute for Brain Research, Peking University, Beijing 100871, China; 6 Beijing Key Laboratory of Behavior and Mental Health, Peking University, Beijing 100871, China; 7 Key Laboratory of Machine Perception (Ministry of Education), Peking University, Beijing 100871, China; 8 Beijing Key Laboratory on Drug Dependence Research, China; 9 The State Key Laboratory of Natural and Biomimetic Drugs, Beijing, China; 10 Key Laboratory for Neuroscience of the Ministry of Education and Ministry of Public Healthy, Beijing, China

## Abstract

Jealousy is not only a way of experiencing love but also a stabilizer of romantic relationships, although morbid romantic jealousy is maladaptive. Being engaged in a formal romantic relationship can tune one’s romantic jealousy towards a specific target. Little is known about how the human brain processes romantic jealousy by now. Here, by combining scenario-based imagination and functional MRI, we investigated the behavioral and neural correlates of romantic jealousy and their development across stages (before vs. after being in a formal relationship). Romantic jealousy scenarios elicited activations primarily in the basal ganglia (BG) across stages, and were significantly higher after the relationship was established in both the behavioral rating and BG activation. The intensity of romantic jealousy was related to the intensity of romantic happiness, which mainly correlated with ventral medial prefrontal cortex activation. The increase in jealousy across stages was associated with the tendency for interpersonal aggression. These results bridge the gap between the theoretical conceptualization of romantic jealousy and its neural correlates and shed light on the dynamic changes in jealousy.

Jealousy is a fundamental social emotion composed of affective, cognitive, and behavioral components^1^. Although jealousy in general could be the result of many different kinds of social comparisons (i.e., social status, wealth, and achievement), romantic jealousy is the most prevalent and important form, as romantic love is a universal human phenomenon and is related to reproduction^2^. Appropriate jealousy, indicating the intention to protect the relationship, is essential for experiencing love and maintaining romantic relationship stability^3^. However, when jealousy goes to the extreme, it can confer tremendous economic and psychological costs on individuals and society, leading to aggressive behaviors such as domestic violence, suicides, and murders^4^,^5^. Romantic jealousy is connected with several psychoses such as substance abuse and affective disorders^6^,^7^. To date, however, we know little about the neural correlates of romantic jealousy.

The affective and experiential core of romantic jealousy is a mixture of some basic emotions, such as anger, sadness and surprise, which arises from a relationship-threatening event^8^,^9^. According to the appraisal theory^10^, emotion, especially complex social emotions, depends on the cognitive apprehension of the antecedents of the emotion. In the case of romantic jealousy, one of the most important antecedents is how one perceives his/her relationship: how one cares for the relationship and what one expects from it^8^. Drawing on a recent theoretical framework that treats social emotion (e.g., guilt, anger) as violation of goal-error^11^, we envision romantic jealousy as a violation of what one expects from a romantic relationship and the romantic partner (e.g., loyal to each other, the goal of living together, etc.). Such expectancy may be reflected in romantic happiness, as those who are happier in their romantic relationship is less likely to believe that their relationship could be affected by a romantic rival. It is thus conceivable that when facing the same relationship-threatening event, those who are happier in their relationship will find it more surprising and unacceptable, and will feel more jealousy. This is a testable hypothesis (Hypothesis 1) to be addressed in this study.

Romantic happiness and jealousy unfold in time. Being engaged in a formal romantic relationship can change romantic jealousy from the initial desire to obtain what one does not have to the fear of losing what one already has^4^,^12^. In a more fine-grained psychological conception, these two stages of jealousy involve similar but not identical feelings^13^. Recently, an online survey of social media found that romantic love and jealousy is elevated after the establishment of romantic relationship^14^. Thus, we hypothesize that being engaged in a formal relationship would result in greater romantic happiness and jealousy than before being engaged in the relationship (Hypothesis 2). Moreover, given the critical role of jealousy-evoked aggression and impulsiveness in response to adverse events, we further hypothesize that the increase in romantic jealousy would be related to individual’s aggressive tendency in relationship (Hypothesis 3).

To test these hypotheses, we investigated the behavioral and neural correlates of romantic jealousy and happiness and their development across stages by combining scenario-based imagination and functional magnetic resonance imaging (fMRI). Forty-two scenarios of romantic relationship (e.g., watching romantic movies, dancing at party) were used to elicit and measure jealousy or happiness. There were four individuals in a scenario: the protagonist (the participant), his/her romantic Partner (with the opposite sex of the participant), the protagonist’s romantic rival (Competitor), and a non-romantic friend (Control, with the same sex as the partner) of both the protagonist and the rival. Jealousy scenarios and the corresponding control scenarios were those depicting the Competitor interacting with the Partner and with the Control, respectively. Happiness scenarios and happiness control scenarios were those depicting the participant interacting with the Partner and the Control, respectively. Each participant underwent two scanning runs (i.e. for before vs. after being in a formal relationship, respectively). Each trial comprised two steps: a scenario-imagining period, during which the participant was asked to read the scenario and imagine the situation vividly without any motor response, and an emotion self-report period, during which the participant move an arrow on a Likert scale to indicate the intensity of jealousy or happiness he/she felt towards the situation. The details are described in the Method and Fig. 1a. We also assessed the association between the changes of romantic jealousy and the aggressive behavioral tendency.

## Results

### Behavioral results

To test Hypothesis 1, we carried out analysis for the correlation between romantic jealousy effect (Partner – Control) and romantic happiness effect (Partner – Control). Confirming our hypothesis, this analysis revealed significant positive correlations between these two effects in both Stage 1 (*r* = 0.61, *P* < 0.001) and Stage 2 (*r* = 0.34, *P* = 0.037) (Fig. 2a,b), suggesting that those individuals who experienced greater happiness with their partner also experienced greater jealousy when they found out that their partners were in intimate encounters with a romantic rival. Moreover, the jealousy effect in both stages positively correlated with the participants’ trait jealousy measured by the Self-report Jealousy Scale^15^ (*r* = 0.44, *P* = 0.006, for Stage 1; *r* = 0.37, *P* = 0.024, for Stage 2; see Fig. S1 for scatter plots). This confirmed the validity of the scenarios and the jealousy rating.

We then carried out a two-way repeated-measures ANOVA for the jealousy ratings, which showed a significant interaction between Target (Partner vs. Control) and Stage (before vs. after being in a formal relationship), *F*_(1,36)_ = 29.10, *P* < 0.001 (Fig. 3a). Specifically, pairwise comparisons showed that from Stage 1 to Stage 2 the jealousy ratings towards the partner significantly increased (*F*_(1,36)_ = 6.20, *P* = 0.018), while the jealousy ratings towards the control significantly decreased (*F*_(1,36)_ = 23.08, *P* < 0.001), thus confirming Hypothesis 2. There was no significant Target by Stage interaction for happiness score (Fig. S2). Moreover, the stage difference for the romantic jealousy effect [(Stage 2_Partner − Stage 2_Control) − (Stage 1_Partner − Stage1_Control)] correlated with the stage differences for the romantic happiness effect, *r* = 0.355, *P* = 0.031 (Fig. 3b).

Finally, we tested the relationship between romantic jealousy and aggressive tendency in romantic relationship. Correlation analysis showed that the increase of jealousy effect from Stage 1 to Stage 2 significantly correlated with scores on the Modified Overt Aggression Scale (Fig. 3c, r = 0.342, *P* = 0.038), indicating that the individuals who were more sensitive to romantic jealousy after being engaged in a formal relationship were more likely to commit interpersonal aggression in romantic relationship (Hypothesis 3).

### Neuroimaging results

#### The romantic jealousy- and happiness-related brain activations

The contrast corresponding to the main effect of romantic jealousy (jealousy event: Partner > Control) produced activations in the basal ganglia (BG), thalamus, middle cingulate, and some other regions (Table 1 and Fig. 4). A parametric analysis with jealousy rating of each trial (see Method) revealed an activation pattern similar to the contrast (see Fig. S3). However, our further analyses and interpretations primarily relied on the contrast results because, as can be seen from Fig. 3a, the emotion ratings clustered around two separate centers (about 2.0 and 5.5) by the Partner/Control manipulation. Using such behavioral data as parametric predictor would violate the prerequisite for correlation analysis. Romantic happiness (happiness event: Partner > Control) produced activations in the medial prefrontal cortex and the posterior midline structures (e.g., precuneus, posterior cingulate cortex; Table 1 and Fig. 5).

#### Stage differences in romantic jealousy-related brain activations

Compared with Stage 1, the jealousy-related brain areas identified in the main effect of jealousy (e.g., the globus pallidus (GP) and the ventral striatum (VS)) showed stronger differential activation (Partner – Control) in Stage 2 (Table 1 and Fig. 6a), which mirrored the pattern of the romantic jealousy ratings. Two-way ANOVA revealed that the regional parameter estimates of the left globus pallidus and ventral striatum showed significant Stage-by-Target interactions, *F*_(1, 36)_ = 8.86, *P* = 0.005 for the left GP, and *F*_(1,36)_ = 6.78, *P* = 0.013 for the left VS) (Fig. 6b,c). As can be seen from the figure, for both of these ROIs, the differential activations between Partner and Control were larger in Stage 2 than in Stage 1, consistent with the finding in jealousy rating.

## Discussion

To our knowledge, this is the first study to explore the dynamic features of romantic jealousy and happiness, i.e., how the change in romantic stages modulates the psychological, behavioral, and neural processes related to romantic jealousy and happiness. We found that BG, especially GP and VS, were the main areas recruited in response to romantic jealousy, and vmPFC was the main area elicited by romantic happiness. Romantic jealousy was significantly affected by romantic happiness. Along with an increase in feelings of romantic jealousy after, relative to before, being engaged in a formal romantic relationship, the jealousy-related activations in the BG were also significantly higher after being involved in a formal romantic relationship. In addition, the increased jealousy was predictive of the level of possible aggression induced by jealousy-related life events.

Intuitively, romantic love has an experiential part that is a positive affective feeling. Closer examination of such concept may reveal a complex and rich phenomenological structure: it may have eudaimonic (e.g., leading a meaningful life with the beloved one), hedonic (e.g., sexual desire, affiliative affect), and mnemonic (e.g., shared memory of past events) aspects^16^. Previous neuroimaging studies of romantic love have predominantly used the picture of the beloved one as stimuli and consistently implicated the striatal areas and temporal cortex (for a meta-analysis, see Cacioppo *et al*.^17^). Here, we used interpersonal interactive events as emotion elicitor, which emphasized more on the evaluation of life events and less on sexual desire or physical attractiveness. We found that vmPFC, an area consistently implicated in social and economic valuation^18^, was strongly activated by happiness-related scenarios involving one’s romantic partner. This may be interpreted as showing that participants assign high value and meaning to the interaction with the beloved ones, consistent with previous studies concerning positive social value and meaning^19^. Future work that applies more clearly defined value function to romantic love could be conducted to investigate the cognitive, affective and computational roles that vmPFC plays in experiencing romantic love.

Romantic jealousy can be seen as a motivated emotion driven by the violation of the social expectation for romantic closeness and loyalty. In social affective domains, the BG is found to play an important role in processing affective consequences of social comparisons^20^ and emotional infidelity in love^21^. Moreover, the GP and VS (i.e., two key components of BG) have been implicated in the processing of expectancy violation in social reinforcement learning^22^,^23^. The VS is activated when viewing the face of those who rejects one’s romantic advancements^24^. These findings indicate the BG may also mediate emotions required for maintaining romantic relationships.

We found stage differences for romantic jealousy in both the behavior and brain activation patterns. After engaging in a formal romantic relationship, individuals may have increased expectancy for loyalty and closeness, rendering the violations more salient when threats occur. In line with this postulate, relative to the first stage, the self-reported intensity of romantic jealousy and the jealousy-related activations in GP and VS increased in Stage 2. Specifically, the stage changes were reflected in the decrease of jealousy in control conditions from Stage 1 to Stage 2 (Figs 3a and 6b,c). This suggests that being engaged in a formal relationship narrows down romantic concern to the relationship partner, which may demonstrate the neural basis of human monogamy.

The increased romantic jealousy after establishing the formal romantic relationship correlated with a higher possibility of interpersonal aggression. According to a population-based survey in China in ref. 25, 7.2% of women aged between 20 and 49 reported that they suffered from partner violence in the past year and that jealousy was a vital source of this risk^26^. Our finding suggests that the degree of increased romantic jealousy across stages may be a predictor for severe violence and aggressive behavior of the intimate partner.

Complex social emotions like romantic jealousy and happiness can be analyzed with a wide range of resolution. For example, at the neurobiological level we can talk about the brain structures or networks that correlates with romantic love and jealousy^17^,^27^, but we can also talk about the specific antecedences and behavioral consequences of those emotions, at the experiential and phenomenological levels^28^. Depending on the levels of analysis, these emotions have different ontological status. At the neural representation level, we share the opinion of many researchers that jealousy, like many other complex social emotions, is not a *sui generis*; rather, it is a specific combination of basic emotions, such as anger and sadness, elicited by specific social stimuli and in specific interactive context^29^. It is thus helpful to view jealousy as secondary emotional processes with evolutionarily well-prepared brain affective substrates, not as an unique process that is genetically ingrained in the inherited neural circuitry^8^. Further studies may be conducted to investigate at the neural level how such a complex emotion can be distinguished from other social emotions such as indignation, guilt, embarrassment, and even its close neighbor envy^30^ and how the basic emotions such as anger and sadness contributed to this complex emotion.

In conclusion, we found that the basal ganglia nucleus encodes romantic jealousy. This neural representation, along with the subjective feeling of jealousy, is modulated by romantic happiness, which correlated with the vmPFC activity, and the stage of the romantic relationship (i.e., before and after being engaged in a formal relationship). Such sharpening effect not only influences jealousy, but also has bearing on violence and aggressive behavior in romantic relationships. Our findings may shed light on the neural underpinning of dynamic changes in jealousy and on the intervention of familial and societal problems related to romantic jealousy.

## Methods

### Participants

Forty right-handed and heterosexual undergraduate students (20 female; aged 23.0 ± 2.1 years, range = 20–29) were recruited by advertisements on campus. Three participants (one male and two females) were excluded from the analysis because of excessive head movements,resulting in a final sample of 37 participants (aged 22.8 ± 1.9 years). All participants, although unmarried, had experienced romantic love and competition according to self-reports. Exclusion criteria included psychiatric disorders, serious physical illness, neurological disorders, alcohol or drug dependence, head injury, or any other contraindications for fMRI scanning. Each individual signed a written informed consent and was paid for their participation. This study protocol was approved by the Peking University Institutional Review Board, and the experiment was carried out in accordance with the approved guidelines.

### Procedure

Before the experiment, participants completed a survey of demographic information and their past and present romantic relationship(s) (i.e., romantic stage, times in a relationship, duration of relationship) in addition to several scales (see below). Next, participants were given detailed instructions for Scenario imagination and task requirements. Each participant was presented with several exemplar scenarios during which happiness or jealousy was assessed. Participants then entered the scanner and completed the fMRI experiment. After scanning, participants were interviewed to better understand their ratings during the scanning session.

### Self-report instruments

All the participants were required to complete a set of self-report instruments after scanning, including the Love Attitude Scale^31^, Experiences in Close Relationships Inventory (ECR)^32^, Self-report Jealousy Scale^15^, and Barratt Impulsiveness Scale-11^33^. Specially, we assessed the level of possible aggression in romantic relationship using Modified Overt Aggression Scale^34^ (including verbal aggression, physical aggression against objects and other people, and autoaggression). In this process, participants were required to evaluate their level of aggression when imagining extreme romantic jealousy. Detailed information about these instruments is provided in the supplementary document. The demographic and instrument data was shown in Table S2.

### The scenario imagination task

#### Characters in scenarios

**Figure 1:**
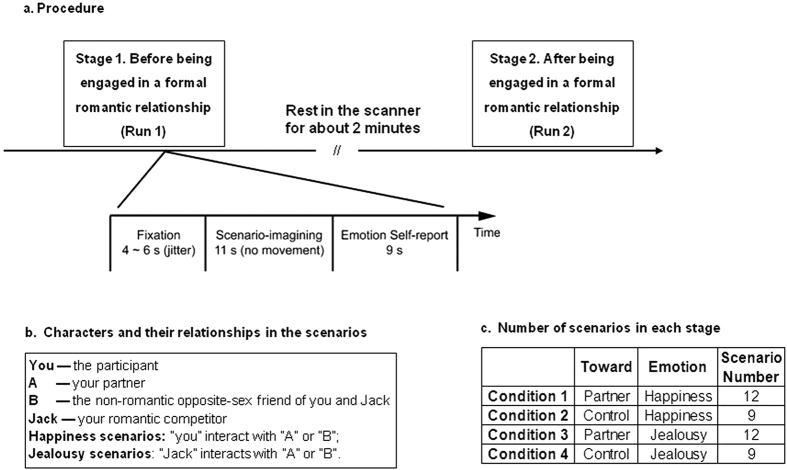
Schematic diagram of the experimental design. (**a**) Each participant underwent two scanning stages, each containing 42 trials. There were two periods in every trial: scenario-imagining period (11 s) and emotion self-report period (9 s). Rating was carried out on a 7-point Likert scale. (**b**) There were four individuals involved in a scenario. For male participants, “A” was a female friend whom both the participant and another male student, “Jack”, liked very much. “B” was a non-romantic mutual friend of the participant and “Jack.” Jealousy scenarios and their corresponding control scenarios were those depicting “Jack” interacting with A or B, respectively. Happiness scenarios and their corresponding control scenarios were those depicting the participant interacting with A or B, respectively. (**c**) Each stage consisted of four conditions presented in randomized order, including happiness related to the partner or the control, and jealousy related to the partner or the control. The number of scenarios in each condition is shown in the table.

**Figure 2:**
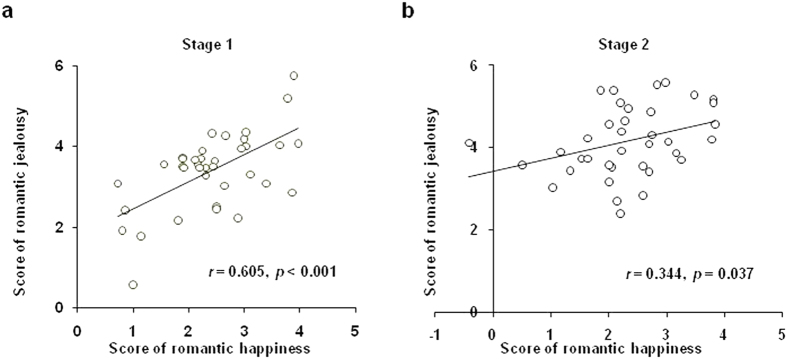
The correlation between romantic jealousy and happiness effects in both Stages. Correlation analysis revealed significant positive correlations between romantic jealousy effect (Partner – Control) and romantic happiness effect (Partner – Control) both in Stage 1 (r = 0.61, *P* < 0.001, **a**) and Stage 2 (r = 0.34, *P* = 0.037, **b**).

**Figure 3:**
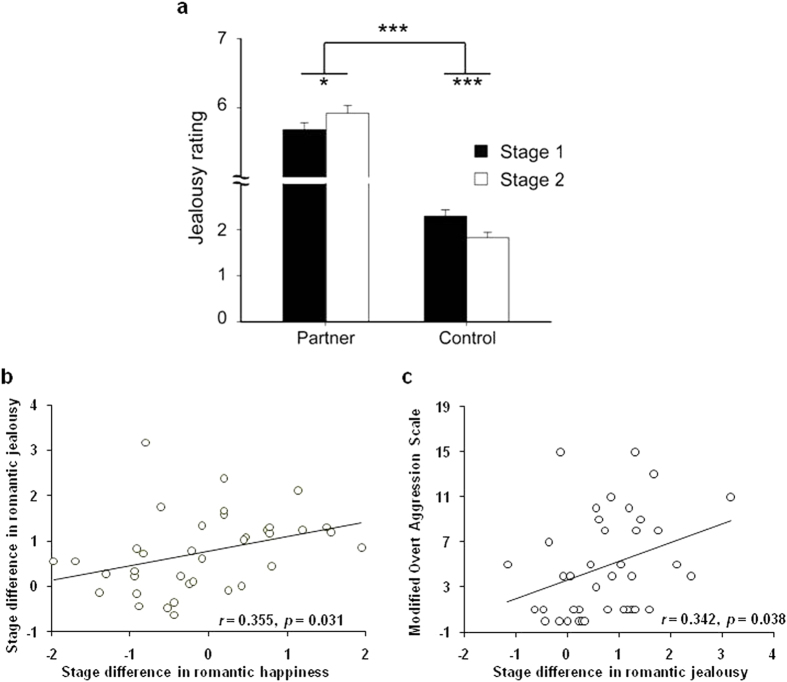
The stage differences of romantic jealousy. (**a**) There was a significant Target (Partner vs. Control) by Stage (Stage 1 vs. Stage 2) interaction for the jealousy rating. (**b,c**) The stage differences of romantic jealousy [(Stage 2_Partner − Stage 2_Control) − (Stage 1_Partner − Stage1_Control)] positively correlated with the stage differences of romantic happiness (**b**) and aggressive tendency as measured by the Modified Overt Aggression Scale (**c**). Error bars indicate standard error. *P < 0.05, ***P < 0.001.

**Figure 4:**
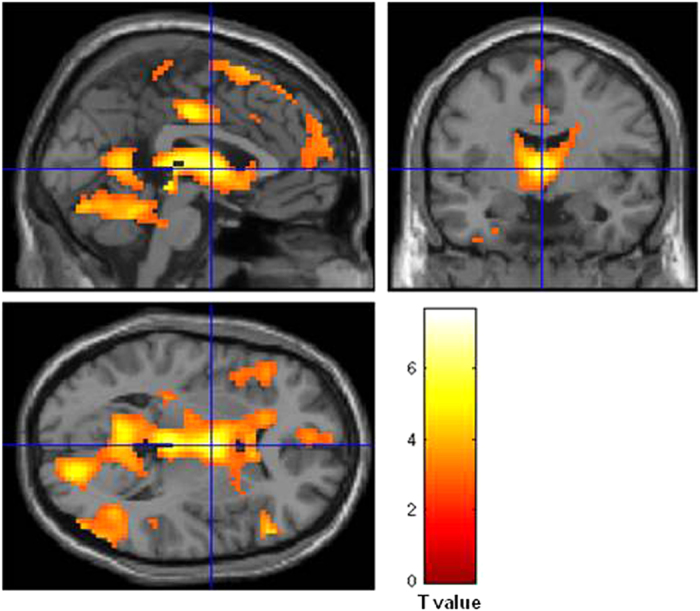
The romantic jealousy-related brain activations. The main effect of romantic jealousy (Partner > Control) produced activations in the basal ganglia, thalamus, middle cingulate, and others.

**Figure 5:**
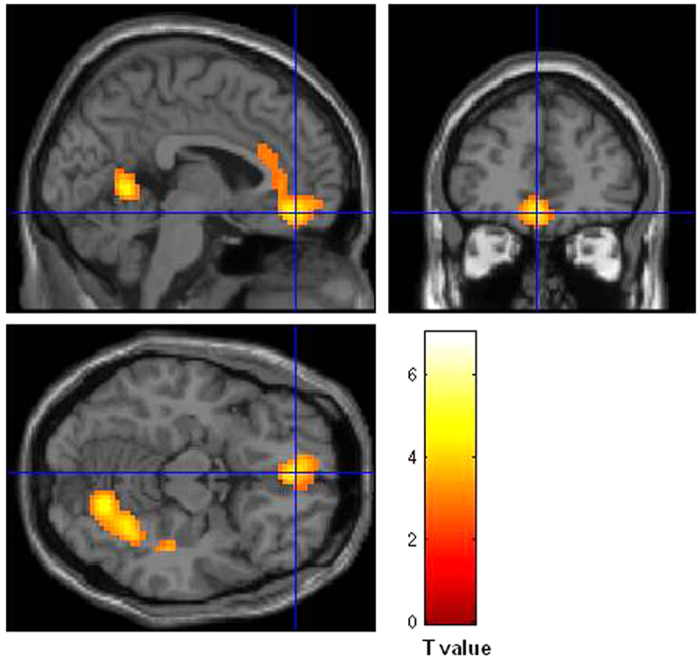
The romantic happiness-related brain activations. Romantic happiness (Partner > Control) produced activations in the medial prefrontal cortex and the posterior midline structures.

**Figure 6:**
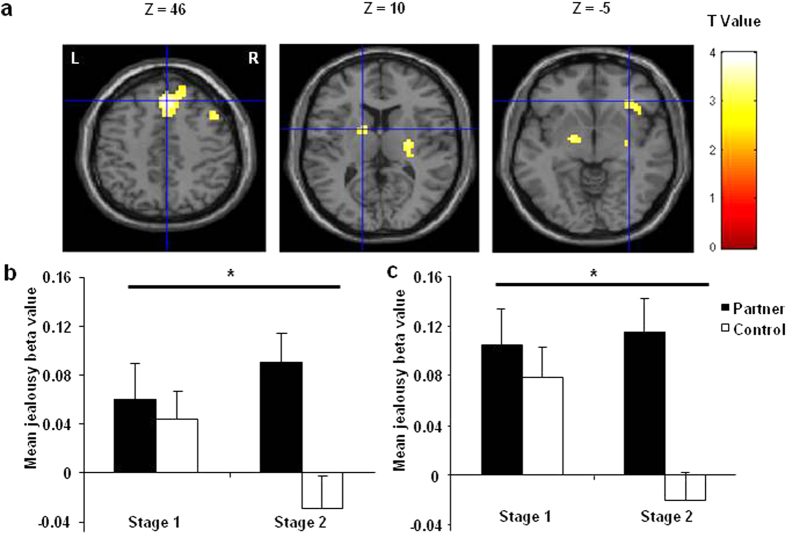
The stage differences of the neural processing of romantic jealousy. (**a**) The romantic jealousy related activation (Partner – Control) significantly increased in Stage 2. (**b,c**) The regional beta weights of the left globus pallidus and ventral striatum (independently defined regions of interest) showing significant Stage-by-Target interaction. Error bars indicate standard error. *P < 0.05.

For male participants, a scenario consisted of four individuals: the participant, the participant’s partner (“A”-partner, opposite sex), the participant’s non-romantic friend (“B”-control, opposite sex), and the participant’s same-sex romantic rival (“Jack”- rival). “A” was a female whom the participant and “Jack” both liked very much. “B” was a non-romantic female mutual friend of the participant and “Jack”. Jealousy scenarios were those depicting “Jack” interacting with A or B. Happiness scenarios were those depicting the participant interacting with A or B (Fig. 1b). Female participants viewed the same scenarios except that the characters in the scenarios were replaced with the opposite sex.

#### Hypothetical scenarios

Forty-two scenarios of romantic relationship were used to measure jealousy or happiness. To better evoke realistic, life-like emotions in the undergraduate participants, scenarios were based on romantic relationships in a campus setting (Supplementary Table S1). These scenarios were assessed prior to experiments by an independent sample (n = 12) and each scenario effectively evoked happiness or jealousy (mean rating > 4 on a 7-point Likert scale ranging from 1-not at all to 7-very strongly).

#### Stages and conditions

Participants underwent two scanning runs. In the first run, participants were required to imagine that A had not shown any preference between the participant himself and Jack (i.e., Stage 1). In the second run, participants were asked to imagine that A and the participant himself had already been in a formal romantic relationship (i.e., Stage 2). To control for the effects of relationship duration on romantic love^35^, participants were required to imagine the scenarios of Stage 2 as being in the early formation of romantic relationship. Between the two runs, participants had roughly two minutes to rest and to prepare for Stage 2.

We created four sets of scenarios, i.e. the four conditions in each Stage [Happiness_Partner (12 scenarios), Happiness_Control (9 scenarios), Jealousy_Partner (12 scenarios), Jealousy_Control (9 scenarios)]. Each condition consisted of distinct scenarios and each scenario appeared with the same frequency across the experiment. The scenarios of four conditions were randomly distributed in each Stage (Fig. 1c).

#### Scenarios imagination process

Participants were required to read each scenario and imagine him/herself as the protagonist and to rate how he/she felt if found in the situation described. To maximally dissociate the neural responses induced by emotion processing and those induced by motor response, we separated the two tasks in every trial: 1) scenario-imagining period: during this period the scenario was presented for 11 seconds and the participants were asked to imagine the scenario and experience the emotions; 2) emotion self-report period: after 11 s of the scenario-imagining period the participants rated their jealousy (in jealousy and jealousy-control conditions) or happiness (in happiness and happiness-control conditions) within 9 s. Rating was carried out on a 7-point Likert scale. The participants moved an arrow on the scale to indicate score they wanted by pressing the left and right buttons using their right index and middle fingers, and then pressed the “confirm” key using the left index finger. The initial arrow appeared equally often on the left and right ends of the scale. The inter-trial interval was 4–6 s, during which the participants were required to fixate on the central dot on the screen (Fig. 1a).

### fMRI data acquisition

Images were acquired using a GE-MR750 3.0 Tesla scanner with a standard 8-channel head coil. The scanning included functional and anatomical imaging. T2*-weighted functional images were acquired, in an interleaved manner, in 40 axial slices parallel to the AC-PC line with no interslice gap, affording full-brain coverage. Images were acquired using an EPI pulse sequence, with a TR of 2000 ms, a TE of 30 ms, a flip angle of 90°, an FOV of 192 mm × 192 mm, and 3 mm × 3 mm × 3 mm voxels. A high-resolution, whole-brain structural scan (1 mm^3^ isotropic voxel MPRAGE) was acquired after functional imaging.

### fMRI data analysis

Image preprocessing and analysis was conducted with the Statistical Parametric Mapping software SPM8 (Wellcome Trust Department of Cognitive Neurology, London, UK). Images were slice-time corrected, motion corrected, resampled to 3 × 3 × 3 isotropic voxel, normalized to the MNI (Montreal Neurological Institute) space, and spatially smoothed using an 8-mm FWHM Gaussian kernel, and temporally filtered using a high-past filter with 1/128 Hz cutoff frequency. The selection of the spatial filer can affect the extent of activation cluster. The parameters we adopted are quite common in the fMRI literature (Takahashi *et al*.^21^). To guarantee that our main findings are not due to the specific smoothing parameters, we also analyzed the data with a 6-mm FWHM Gaussian kernel in spatial smoothing. It turned out that, our major imaging results remained when we use the 6-mm FWHM Gaussian kernel. Therefore, we report only the neuroimaging findings derived from the 8 mm smoothing kernel. Correction for temporal autocorrelations using AR(1) was also carried out.

In the first-level (within-participant) analysis, we defined a factorial model and a parametric model. Our data analysis focused on the emotion-evoked period. For the factorial analysis, we modeled the emotion-evoked period (11 s) using four regressors, each corresponding to one experimental condition. An additional regressor was used to model the response period. For the parametric analysis, all scenario-reading events corresponding to the jealousy content (i.e., scenarios of the jealousy-partner and jealousy-control conditions in both Stages) were combined into a single regressor. The jealousy rating of each trial was added to this regressor as a first-order parametric modulation. Similarly, all scenario-reading events corresponding to the happiness content (i.e., scenarios of the happiness-partner and happiness-control conditions in both Stages) were combined into a single regressor, with the happiness rating as the first-order parametric modulation. For both the factorial and the parametric analysis, events were modeled with boxcar regressors (duration = 11 s) convolved with standard hemodynamic response function (HRF). The six rigid body parameters were included to account for head motion artifacts. Based on these first level analyses, we carried out the second (group) level analyses both within predefined regions-of-interest and on the whole-brain. For the ROI analysis, we extracted the parameter estimates (beta value) around the coordinates reported in previous studies on jealousy (left GP, *x* = −12, *y* = 2, *z* = 2^21^, left VS, *x* = −7, *y* = 12, *z* = −4^20^, and left vmPFC, *x* = −3, *y* = 44, *z* = −15^36^). Parameter estimates were extracted from a cube (each side length of the cube was 3 voxels) containing 27 voxels around these coordinates and subject to a 2 Stages (before vs. after being in a formal relationship) by 2 Targets (Partner vs. Control) repeated-measures ANOVA, which separately did for happiness and jealousy. At the whole-brain level, the factorial analysis and the parametric analysis were carried out separately. For the factorial analysis of each emotion, the four individual contrast maps corresponding to the presentation of scenarios were fed into a flexible-factorial matrix, i.e., “Partner_Before”, “Control_Before”, “Partner_After”, and “Control_After”. We defined 2 sets of contrasts: (1) the main effect of target: (“Partner_Before” + “Partner_After”) − (“Control_Before” + “Control_After”) and (2) the effect of stage on emotion: (“Partner_After” − “Control_After”) − (“Partner_Before” − “Control_Before”). For the parametric analysis, individual beta maps corresponding to the interaction term (rating-by-target) were fed into a one-sample t-test model (random-effect analysis).

The statistical threshold of imaging data was *P* < 0.05 at cluster-level using family-wise error correction (*P* < 0.005 uncorrected at voxel-level with a cluster size > 46 voxels, which was determined by AlphaSim program (http://afni.nih.gov/afni/docpdf/AlphaSim.pdf).

## Additional Information

**How to cite this article**: Sun, Y. *et al*. Neural substrates and behavioral profiles of romantic jealousy and its temporal dynamics. *Sci. Rep.* **6**, 27469; doi: 10.1038/srep27469 (2016).

## Supplementary Material

Supplementary Information

## Figures and Tables

**Table 1:** Brain areas revealed by whole-brain contrasts (factorial analysis).

**Order**	**Regions**	**Hemi**	**Max** ***T*****-value**	**Cluster size (voxels)**	**MNI Coordinates**		
					***x***	***y***	***z***
Romantic Jealousy							
1	Lingual Gyrus	R	7.67	5270	12	−76	−14
	Occipital Fusiform	R	7.37		18	−70	−17
	Cerebellum	R	6.55		20	−67	−23
2	Cingulate Gyrus, posterior division	L	6.08	200	−3	−16	40
	Precentral Gyrus	L	3.22		−30	−28	43
	Postcentral Gyrus	L	3.15		−18	−37	46
3	Superior Frontal Gyrus	L	5.1	936	−6	17	67
	Frontal Pole	L	4.27		9	59	34
	Frontal Pole	L	4.24		−6	65	16
4	Inferior Frontal Gyrus	R	4.41	63	54	29	4
5	Frontal Orbital Cortex	L	3.74	262	−39	29	−2
	Inferior Frontal Gyrus, pars triangularis	L	3.55		−45	29	7
	Frontal Operculum Cortex	L	3.4		−45	14	1
6	Postcentral Gyrus	L	3.21	61	−15	−34	67
	Precentral Gyrus	L	3.2		−3	−31	67
Stage Differences in Romantic Jealousy							
1	Superior Frontal Gyrus	R	3.99	279	3	32	46
	Superior Frontal Gyrus	R	3.62		0	20	52
	Frontal Pole	R	3.26		24	53	37
2	Middle Frontal Gyrus	R	3.95	53	45	17	40
3	Pallidum	L	3.66	60	−18	−7	−2
	Caudate	L	3.03		−15	2	10
4	Pallidum	R	3.38	83	27	−13	10
	Thalamus	R	3.14		18	−16	19
	Pallidum	R	2.78		30	−10	−5
5	Frontal Orbital Cortex	R	3.3	94	42	29	−11
	Frontal Orbital Cortex	R	3.18		33	29	−5
	Frontal Orbital Cortex	R	2.76		24	26	−11
Romantic Happiness							
1	Lingual Gyrus	R	7.01	1354	15	−70	−8
	Lingual Gyrus	L	5.43		−12	−55	4
	Precuneous Cortex	L	5.04		−12	−58	13
2	Lateral Occipital Cortex	L	6.2	103	−42	−79	31
3	Frontal Medial Cortex	L	4.37	276	−3	41	−14
	Cingulate Gyrus, anterior division	L	3.63		0	35	4
	Cingulate Gyrus, anterior division	L	3.01		−6	26	19
4	Central Opercular Cortex	R	4.17	155	54	−10	16
	Supramarginal Gyrus, anterior division	R	3.47		69	−22	19
	Supramarginal Gyrus, anterior division	R	3.46		60	−19	22

The statistical threshold was set at *P* < 0.05 FWE-corrected for multiple comparison (*P* < 0.005 with a cluster size > 46 voxels).
