# Supplementary material for: Neural substrates and behavioral profiles of romantic jealousy and its temporal dynamics
Source: Sci Rep. 2016 Jun 7;6:27469. doi: 10.1038/srep27469 (PMC4895349; doi:10.1038/srep27469)
Supplement: Supplementary Information [file srep27469-s1.doc]

Supplementary Material for *Scientific Reports*

**Neural substrates and behavioral profiles of romantic jealousy and its temporal dynamics**

Yan Sun^1,+^, Hongbo Yu^2,+^, Jie Chen^1,3,+^, Jie Liang^1,3^, Lin Lu^1,4,5^, Xiaolin Zhou^2,5,6,7^,*, Jie Shi^1,8,9,10^,*

*^1^National Institute on Drug Dependence, Peking University, Beijing 100191, China.*

*^2^Center for Brain and Cognitive Sciences and Department of Psychology, Peking University, Beijing 100871, China.*

*^3^Department of Pharmacology, School of Basic Medical Sciences, Peking University Health Science Center, Beijing 100191, China.*

*^4^Institute of Mental Health/Peking University Sixth Hospital and National Clinical Research Center for Mental Disorders & Key Laboratory of Mental Health, Peking University, Beijing 100191, China.*

*^5^Peking-Tsinghua Center for Life Sciences and PKU-IDG/McGovern Institute for Brain Research, Peking University, Beijing 100871, China.*

*^6^Beijing Key Laboratory of Behavior and Mental Health, Peking University, Beijing 100871, China**.*

*^7^Key Laboratory of Machine Perception (Ministry of Education), Peking University, Beijing 100871, China.*

*^8^Beijing Key Laboratory on Drug Dependence Research, China.*

*^9^The State Key Laboratory of Natural and Biomimetic Drugs, Beijing, China.*

*^10^Key Laboratory for Neuroscience of the Ministry of Education and Ministry of Public Healthy, Beijing, China.*

*To whom correspondence should be addressed.

^+^ These authors contributed equally to this work.

**This file includes:**

Supplementary methods

Supplementary Fig. S1-S3

Supplementary Table S1-S2

*Corresponding authors:

**Prof. Jie Shi**

National Institute on Drug Dependence, Peking University, 38 Xue Yuan Road, Beijing 100191, China

E-mail: shijie@bjmu.edu.cn

Tel: +86-10-82801593/Fax: +86-10-62032624

Or

**Prof. Xiaolin Zhou**

Department of Psychology, Peking University, 5 Yiheyuan Road, Beijing 100871, China

E-mail: xz104@pku.edu.cn

Tel: +86-10- 62756599/Fax: +86-10-62761081

**Supplementary methods**

*Self-Report Instruments*

The Love Attitude Scale (LAS)[^1^](#_ENREF_1). Love attitudes were assessed using the 42-item scale developed by Hendrick and Hendrick (1986), which consists of 7 items measuring each of six kinds of love attitudes (Eros, Ludus, Storge, Pragma, Mania, Agape). Participants appraise each item using a 5-point rating scale of 1(*strongly disagree*) to 5 (*strongly agree*).

Experiences in Close Relationships Inventory (ECR)[^2^](#_ENREF_2). This 36-item questionnair consists of two dimensions: anxiety and avoidance. Each item is rated on a scale of 1 (*strongly disagree*) to 7 (*strongly agree*) to indicate to what extent participants agree with the expression. Higher score on either or both of the two dimensions means more insecure attachment orientation.

Self-report Jealousy Scale[^3^](#_ENREF_3). Self-report jealousy scale consists of 20 scenarios to test if participants feel jealousy in these situations. It is on a scale from 0 (*pleased*) to 4 (*extremely upset*).

Barratt Impulsiveness Scale-11. Barratt Impulsiveness Scale-11(BIS-11) is a gold-standard instrument to assess impulsivity on three different domains: attentional impulsiveness, defined as making quick decisions; motor impulsiveness, defined as acting without thinking and non-planning impulsiveness, defined as a lack of concern for the future[^4^](#_ENREF_4). It includes 30 questions and each one is rated on a scale of 1(*never*) to 4 (*always*). BIS-11 score ranges from 30 to 120 and higher score means the higher level of impulsivity.

Modified Overt Aggression Scale[^5^](#_ENREF_5). It includes verbal aggression, physical aggression against objects and other people and autoaggression. Total MOAS score was obtained by assigning a different weight for each type of aggression.


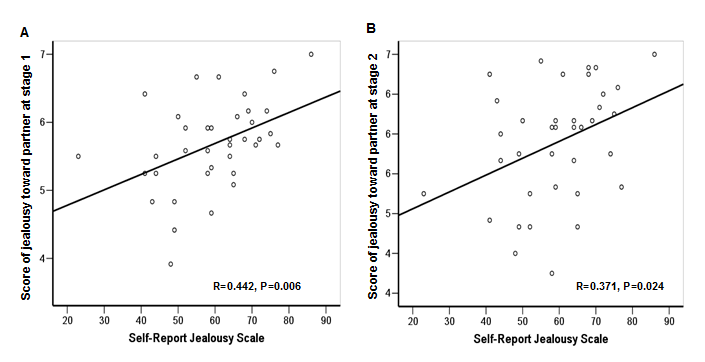


Figure S1. The validity of the jealousy ratings. The jealousy ratings toward partner were positively correlated with the scores of Self-Report Jealousy Scale in Stage 1 (A) and Stage 2 (B), respectively.


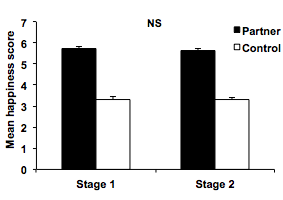


Figure S2. The mean score of happiness. There was no significant Target (Partner vs. Control) by Stage (Stage 1 vs. Stage 2) interaction for happiness score.


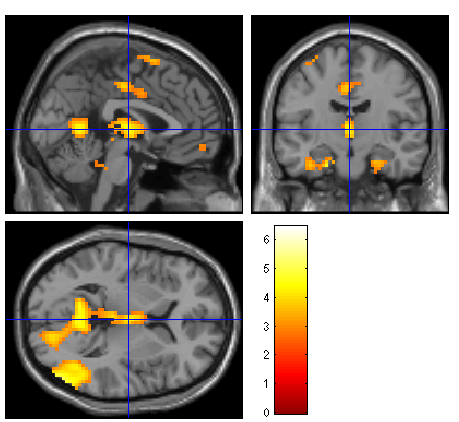


T value

Figure S3. Brain areas whose jealousy effect was positively correlated with romantic jealousy ratings.

Table S1. Scenarios used to assess jealousy and happiness for male participants.

| No. | Scenarios |
| --- | --- |
| 1 | Watch romantic movies |
| 2 | Hear her favorable comments about you/Jack from others |
| 3 | Go shopping |
| 4 | Go to the library to borrow some books |
| 5 | Go on a hike at weekends |
| 6 | She like the gift from Jack/you very much |
| 7 | Send her to some place by bicycle |
| 8 | Sit together at the study hall |
| 9 | Encounter in the playground and jogged together |
| 10 | Accompany and cheer her on when she participate in a competition |
| 11 | Chat over coffee after the gruelling test |
| 12 | Participate in a new year's eve party |
| 13 | Take a walk in the school garden after dinner |
| 14 | Being photographed separately when friends taking a group photo |
| 15 | Help bring the breakfast. |
| 16 | Have many common topics to talk about |
| 17 | Come to visit when fall ill |
| 18 | Have dinner after class |
| 19 | She pours troubles out to you/Jack sometimes |
| 20 | Team up to join in activities or competitions |
| 21 | Go boating together during the class trip |
| 22 | Dance at the party |
| 23 | Send her back to the dormitory after evening classes |
| 24 | Be in the same students' organization and work together |
| 25 | Accompany together when working late for experiments |
| 26 | Go to a concert to watch the performance of her best-loved singer |
| 27 | Take the same train when going hometown |
| 28 | Play cards together |
| 29 | Sing love songs together in the KTV |
| 30 | Chat outside the classroom during breaks |
| 31 | Go to have snacks after evening classes |
| 32 | Accompany together when group travelling |
| 33 | She gives you a call for help |
| 34 | Send messages to care for each other (e.g. to remind the weather changes) |
| 35 | Come to comfort forwardly when in trouble |
| 36 | Pick up her at the station when returning back to school |
| 37 | Watched favorite TV shows |
| 38 | Go to the amusement park |
| 39 | Go to the park at the weekends |
| 40 | Go to a restaurant which she looked forward to for a long time |
| 41 | Walk together under an umbrella when raining. |
| 42 | Interact on social networking sites. |

Female participants received the same scenario except that the gender of the characters and descriptions regarding gender were replaced by the opposite gender.

Table S2. The demographic and personality data.

| Demographic Items | Mean (SD) | Score of Scale | Mean (SD) |
| --- | --- | --- | --- |
| Age (year) | 22.80 (1.89) | Barratt Impulsiveness Scale | 64.95 (6.65) |
| Education (year) | 16.41 (1.64) | Attentional impulsiveness | 17.51 (2.24) |
| Income (￥/month) | 1622 (617) | Motor impulsiveness | 21.59 (2.31) |
| Number of siblings | 1.73 (0.84) | Non-planning impulsiveness | 25.84 (3.86) |
| Birthplace |  | Love Attitudes Scale |  |
| City | 14 (37.8%) | Eros | 25.46 (2.93) |
| Town | 4 (10.8%) | Ludus | 19.68 (4.21) |
| Country | 19 (51.4%) | Storge | 22.03 (3.72) |
| Romantic education of parents |  | Pragma | 25.30 (3.02) |
| Not mention | 8 (21.6%) | Mania | 23.89 (4.22) |
| Let go | 15 (40.5%) | Agape | 24.24 (3.88) |
| Active | 1 (2.7%) | Experiences in Close Relationships Inventory | |
| Tradition | 13 (35.1%) | Attachment avoidance | 53.70 (12.69) |
| Romantic stage |  | Attachment anxiety | 72.92 (12.14) |
| Single, not ambiguous | 4 (10.8%) | Self-report Jealousy Scale | 59.38 (12.78) |
| Single but ambiguous | 9 (24.3%) | Modified Overt Aggression Scale | 4.68 (4.56) |
| Being in a relationship | 24 (64.9%) |  |  |
| Total times in a relationship | 1.38 (0.55) |  |  |
| Total duration in love (month) | 29.50 (20.57) |  |  |

References

1. Hendrick, C., & Hendrick, S. A theory and method of love. *J. Pers. Soc. Psychol.* **50**, 392-402 (1986).

2. Fraley, R.C., Waller, N.G., & Brennan, K.A. An item response theory analysis of self-report measures of adult attachment. *J. Pers. Soc. Psychol.* **78**, 350-365 (2000).

3. Bringle, R.B., Roach, S., Andler, C., & Evenbeck, S. Measuring the intensity of jealous reactions. *JSAG: Catalog of select documents in psychology* **9**, 2324 (1979).

4. Patton, J.H., Stanford, M.S., & Barratt, E.S. Factor structure of the Barratt impulsiveness scale. *J. Clin. Psychol.* **51**, 768-774 (1995).

5. Kay, S.R., Wolkenfeld, F., & Murrill, L.M. Profiles of aggression among psychiatric patients. I. Nature and prevalence. *J. Nerv. Ment. Dis.* **176**, 539-546 (1988).
